# Long-Term Effects of Three Different Appliances for Rapid Maxillary Expansion: A Systematic Review and Meta-Analysis

**DOI:** 10.1016/j.identj.2025.104024

**Published:** 2025-11-20

**Authors:** Jingyi Yu, Feng Qiu, Mengfei Xu, Jixiao Wang, Lan Li, Fulan Wei

**Affiliations:** Department of Orthodontics, School and Hospital of Stomatology, Cheeloo College of Medicine, Shandong University & Shandong Key Laboratory of Oral Tissue Regeneration & Shandong Engineering Research Center of Dental Materials and Oral Tissue Regeneration & Shandong Provincial Clinical Research Center for Oral Diseases, Jinan, China

**Keywords:** Rapid maxillary expansion, Effectiveness, Adverse effects, Systematic review, Meta-analysis

## Abstract

**Objective:**

To compare the long-term effects (≥6 months) of bone-borne (BB) and tooth-BB appliances with tooth-borne (TB) appliances in rapid maxillary expansion.

**Materials and Methods:**

An electronic search was conducted in PubMed, Cochrane Library, Scopus, Embase, Web of Science, Proquest, ClinicalTrials.gov, and OpenGrey. The Grading of Recommendations, Assessment, Development, and Evaluation approach was used to evaluate evidence. Mean differences were calculated, applying a random effects model.

**Results:**

Nine studies were included. Six articles were randomized controlled trials (RCTs), two were retrospective studies, and one was a parallel cohort study. Three non-RCTs were at moderate risk of bias. Three RCTs were at unclear risk of bias, while three were at high risk of bias. This review specifically focused on evidence from three-dimensional assessment methods (CBCT/CT). No difference in dental maxillary expansion and skeletal maxillary expansion between appliances, though TB appliances may cause greater molar inclination in the treatment. No differences in buccal bone resorption were observed over a follow-up period exceeding 1 year.

**Conclusions:**

TB, tooth-BB, BB appliances demonstrated comparable long-term effects regarding the amount of dental and skeletal maxillary expansion, molar inclination, and buccal bone resorption.

## Introduction

Transverse maxillary deficiency is a common clinical malocclusion seen among adolescents and adults, with a prevalence of over 8% to 10%.^1^ It can manifest clinically as a unilateral or bilateral crossbite, crowded teeth, or a narrow nasal cavity.^2^^,^^3^ Transverse maxillary deficiency is frequently treated by rapid maxillary expansion (RME), first reported by Angel in 1860 and later promoted by Haas in the 1960s.^4^^,^^5^ Currently, expansion appliances are generally divided into three types: tooth-borne (TB) appliances, bone-borne (BB) appliances, and tooth-BB (TBB) appliances.

Evidence from past short-term results suggests that common undesirable outcomes of TB appliances include limited skeletal movement, molar inclination, root resorption, and detrimental periodontal effects.^6^, ^7^, ^8^, ^9^ BB appliances may induce more physiologic sutural expansion and reduce negative dental effects.^10^ TBB appliances have been designed to minimize side effects and improve skeletal changes.^11^ However, several included studies had a 3-month observation period and partially relied on measurements from plaster cast models.^10^^,^^12^ Data measured using three-dimensional methods are more accurate and comprehensive.^13^, ^14^, ^15^ Furthermore, clinical evidence about the long-term effect of different appliances has not been systematically and critically appraised.

Thus, the purpose of this study was to compare the effects of TB appliances with BB appliances or TBB appliances 6 months or longer after RME.

## Materials and methods

This systematic review was registered at the International Prospective Register of Systematic Reviews (PROSPERO).

### Eligibility criteria

The PICOS (Participants, Intervention, Comparison, Outcome, Study) principle was used to establish the criteria for selecting studies. These criteria are listed in Table 1. No publication status, language, or year restrictions were applied.

**Table 1:** Inclusion and exclusion criteria used for study selection (PICOS).

Component	Inclusion criteria	Exclusion criteria
**Participants**	1. Growing patients (age under 18 y) with maxillary transverse deficiency treated by nonsurgical rapid maxillary expansion 2. 10 patients or more in each study (as a total number of participants)	1. Patients with cleft lip and palate, craniofacial syndromes, temporomandibular joint disorders, previous orthodontic treatment, or medically compromised patients
**Intervention**	Bone-borne or tooth-bone-borne rapid maxillary expansion	Additional treatment performed besides expansion during study period, like headgear or facemask therapy
**Comparison**	Tooth-borne rapid maxillary expansion.	
**Outcome**	1. Three-dimensional evaluation (CT, CBCT, or MRI) 2. Primary outcomes: dental maxillary expansion 3. Additional outcomes: Skeletal maxillary expansion; buccal bone reduction; molar angulation	1. No baseline measurements 2. The long-term measurements were obtained no more than 6 mo after expansion
**Study design**	1. Randomised clinical trials 2. Quasi-randomised clinical trials 3. Prospective controlled clinical trials 4. Retrospective controlled trials 5. cohort trials	1. Abstracts 2. Comments 3. Case reports 4. Narrative reviews 5. Case series 6. Expert opinion 7. Systematic reviews and meta-analyses 8. In vitro studies

### Search strategy

A comprehensive strategy using a combination of controlled vocabulary (MeSH terms) and free text terms was developed (Supplementary Table 1). The search strategy was modified for each database and implemented on 28 November 2024. Databases searched electronically were PubMed, the Cochrane Library, Scopus, Embase, Web of Science, Proquest, ClinicalTrils.gov. Additionally, a hand search was performed, and the grey literature was searched through a Google Scholar web search.

### Studies selection and data extraction

Following the removal of duplicates, the retrieved records were screened initially by titles and abstracts; later, the remaining articles were assessed by full text. Disagreements were resolved through discussion.

Data extraction was carried out by two authors independently and in duplicate from the studies that had met the inclusion criteria using a customized data collection form. Conflicts, once presented, were resolved by discussion between two authors. Collected data included: type of study, authors, year of publication, clinical settings, country, sample size, gender distribution, mean age, appliances design, activation protocols, and other details about evaluation periods.

### Assessment of risk of bias in individual studies

The risk of bias of included studies was assessed according to Cochrane guidelines with the version 2 of the Revised Cochrane risk-of-bias tool (RoB 2) for randomized controlled trials (RCTs) and the Risk of Bias in Nonrandomized Studies of Interventions tool (ROBINS-I) for non-RCTs.^16^^,^^17^ Two authors assessed the risk of bias independently and in duplicate. Disagreements were resolved during a discussion between two authors.

### Effect measures and synthesis methods

A narrative synthesis of the findings from the included studies was provided. Clinical, methodological, and statistical heterogeneity was evaluated. A quantitative synthesis (meta-analysis) using the Review Manager (RevMan) 5.4.1 software was conducted if the included studies were sufficiently homogeneous. A random effect model was applied. The mean of the differences between treatments was reported for the aggregation of continuous data. Inverse of variance method and a 95% confidence interval were calculated.

Heterogeneity was assessed through chi-square test (in which a *P* value <.1 indicated a statistically significant heterogeneity) and through the inconsistency index (*I*^2^). Values above 50% represented substantial heterogeneity.

The results of the meta-analysis were reported with a forest plot.

### Reporting bias assessment

Risk of bias of included studies were reported graphically with the risk of bias traffic light plot of ROB2 assessments created using robvis.^18^

### Certainty assessment

The certainty of evidence was assessed by the Grading of Recommendations, Assessment, Development, and Evaluation (GRADE).^19^^,^^20^ The following parameters were assessed by two reviewers: RoB, inconsistency (heterogeneity), indirectness, imprecision, and publication bias.^21^, ^22^, ^23^, ^24^, ^25^ The quality of evidence was classified into four levels: high, moderate, low, and very low.

## Results

### Study selection

**Fig. 1:**
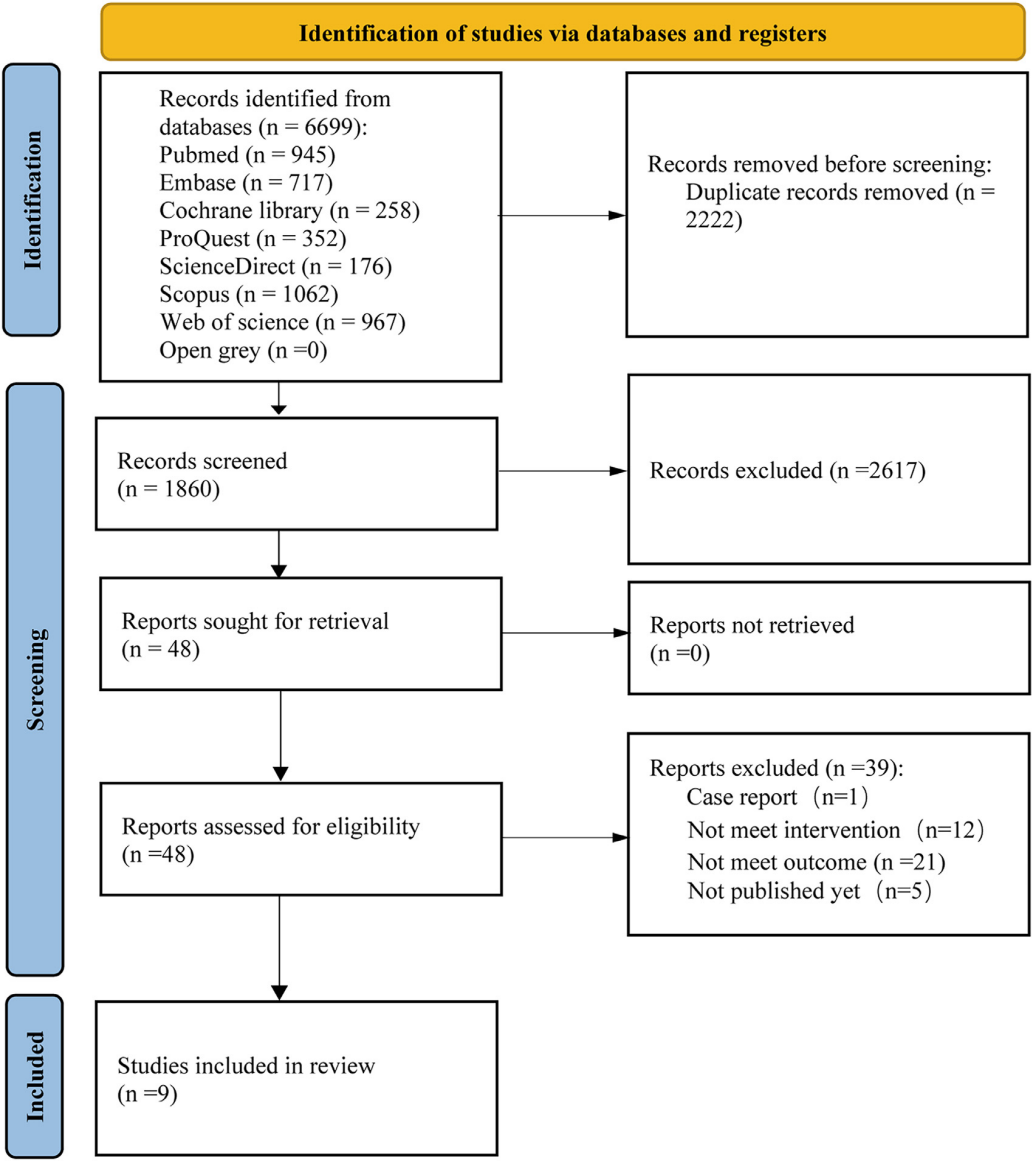
PRISMA 2020 statement flowchart.

The search strategy and selection of the studies are represented in the PRISMA flow diagram (Figure 1). Removal of duplicates and screening by titles and abstracts resulted in a total of 58 articles. Subsequently, 49 records were excluded because of not meeting the eligibility criteria. Finally, nine records (7 data sets) were included.^26^, ^27^, ^28^, ^29^, ^30^, ^31^, ^32^, ^33^, ^34^

Table 2, Table 3 represent the characteristics of the included studies. All of the included studies primarily presented results about dental maxillary expansion. Six studies reported results about skeletal maxillary expansion^26^, ^27^, ^28^, ^29^, ^30^^,^^33^; three studies reported results about buccal bone reduction^26^^,^^31^^,^^33^; and seven studies reported results about molar inclination.^26^, ^27^, ^28^, ^29^, ^30^, ^31^^,^^33^

**Table 2:** Characteristics of included studies.

Study	Setting/country	Study design	Sample size/gender	Mean age	Outcomes
Lagravère et al^28^^,^^42^	Uni, Canada	RCT	62 P; 20 TB (15 F; 5 M); 21 BB (13 F; 8 M); 21 C (15 F; 6 M)	BB: 14.24 ± 1.32 y; TB: 14.05 ± 1.35 y; C: 12.86 ± 1.19 y	IMW(T), IMW(B), MBA16-PC16-PC26, MBA26-PC26-PC16
Bazargani et al^32^^,^^34^	Hospital, Sweden	RCT	52 P; 26 TB (13 F; 13 M); 26 TBB (13 F; 13 M)	8-13 y	IMW(T), TV1, TV2, MBL dx, MBL sin
Mehta et al^26^	Uni, Canada	Retrospective study	60 P; 21 TB; 20 BB; 19 C	BB: 13.69 ± 1.74 y; TB: 13.9 ± 1.14 y; C: 13.3 ± 1.49 y	IMW(T), IMW(B), molar angulation, BTR, BTAC, vertical buccal bone height
Kayalar et al^29^	Uni, Australia	RCT	40 P; 20 TB, (10 F, 10 M); 20 TBB, (12 F, 8 M)	13.86 ± 1.60 y	IMW(T), IMW(B), Inc_26
Celenk-Koca et al^31^	Uni, Canada	RCT	40 P; 20 TB (12 F, 8 M); 20 BB (13 F, 7 M)	TB: 13.84 ± 1.36 y; BB: 13.81 ± 1.2 y	IMW(T), MBLI, PBLI, MBW, PBW
Garib et al^30^	Uni, Brazil	Retrospective study	32 P; 18 TBB (8 F, 10 M); 14 TB (6 F, 8 M)	TBB: 10.80 y; TB: 11.44 y	IMW(T), IMW(B), molar inclination
Altieri and Cassetta^33^	Uni, Rome	Parallel cohort study	26 P; 14 TB (6 F, 8 M); 12 BB (5 F, 7 M)	12.3 ± 0.82 y	IMW(T), IMW(B), BTR, BTAC, MBL, MBR, DBL, DBR, left/right molar angulation

BB, bone borne; BTAC, alveolar bone to the alveolar crest; BTR, alveolar bone to root apex; C, control; DBL, left distobuccal bone plate thickness; DBR, right distobuccal bone plate thickness; F, female; IMW(B), intermolar width (skeletal); IMW(T), intermolar width (dental); Inc, the angle between the palatal cusp and the internal hard palate; MBA, mesial buccal apex; MBL sin, marginal bone level at buccal aspect of right and left first molar, respectively; MBL, left mesiobuccal bone plate thickness; MBLI, buccolingual inclinations of the maxillary first molars; MBR, right mesiobuccal bone plate thickness; MBW, molar buccal width; P, patients; PBLI, buccolingual inclinations of the maxillary first premolars; PBW, premolar buccal width; PC, pulp chamber; RCT, randomized clinical trial; TB, tooth borne; TBB, tooth-bone borne; TV1, TV2, angle of teeth between the line passing through the palatal root apex and palatal cusp at right and left first molar, respectively and the vertical line parallel to the midsagittal plane MBL dx; Uni, university; y, year.

**Table 3:** The activation protocol and data collection of included studies.

Study	Expansion appliance	Expansion protocol	Duration treatment, expansion or retention	Evaluation periods
Lagravère et al^28^	TB: bands on the first permanent molars and first premolars; BB: placed on each side between the projection of the permanent first molars and second premolar.	TB: twice a day (0.25 mm per turn, 0.5 mm daily) BB: 1 turn of the screw every other day	Duration expansion: until posterior dental crossbite overcorrection was achieved. Retention: 6 mo	TB, BB: baseline (T1), after activation of the appliance (T2), after removal of the appliance 6 mo (T3), before fixed bonding (12 mo, T4); C: T1, T3, T4
Bazargani et al^32^^,^^34^	TB: bands on the first molars, and incorporated palatal extensions extending on the palatal side of the upper premolars. TBB: two mini-screw implants attaching the expander to the palate surface; bands on the first molars.	Two quarter turns per day (0.5 mm)	Duration expansion: until the palatal cusps of the maxillary first molars contacted the buccal cusps of the mandibular first molars. Retention: 6 mo	Baseline (T0); after removal of appliance 6 mo (T1); 1 y postexpansion, (T2); 5 y postexpansion (T3)
Mehta et al^26^	TB: bands on the first permanent molars and first premolars. BB: 2 miniscrews placed in the palatal region.	2 turns each day	T1-T3: BB, 2 y 8 mo; TB, 2 y 9 mo; C: 2 y 7 mo	Baseline (T1); postexpansion (T2) in BA and TA groups and 6 mo after initial CBCT scan in the control group; posttreatment (T3)
Kayalar et al^29^	TB: bands on the first premolars and molars. TBB: two mini-screws placed at the level of the third rugae on either side of the midpalatal suture and bands on the first molars, and incorporated palatal extensions extending on the palatal side of the upper premolars.	Twice a day (0.5 mm daily)	Duration expansion: until an overcorrection of 30% was achieved and determined by observing that the palatal cusps of the upper first molars were in contact with the buccal cusps of the lower first molars. Retention: 6 mo	Baseline (T0); after a 6-mo retention period (T1)
Celenk-Koca et al^31^	TB: bands on the premolars and first molars and incorporated palatal extensions. BB: implants were placed between the first and second premolars, and between the second premolars and first molars.	Two turns a day	Duration expansion: 19.7 ± 3.8 d Retention: 6 mo	Baseline (T1); 6 mo (T2) following a passive retention period
Garib et al^30^	TB: bands on the maxillary first permanent molars and bonded C-shape clasps on the maxillary canines or premolars. TBB: bands on the first permanent molars. Two miniscrews were installed in the expander slots.	A one-quarter turn twice a day for 14 d	Duration expansion: until achieving 5.6 mm of expansion. Retention: 11 mo	Baseline (T1); after the expander removal (T2)
Altieri and Cassetta^33^	TB: bands on the maxillary first molars. BB: 4 miniscrews inserted both in the paramedian and parapalatal position of the palatal vault.	4 quarter turns on the first day and by 3 quarter turns per day in the active phase of treatment (0.20 mm per turn, 0.6 mm daily)	Duration expansion: until an 8 mm screw opening was achieved Retention: 6 mo	Baseline (T1); posttreatment stage (T2), at the 6-mo retention period

mm, millimetre.

### Risk of bias within the studies

**Fig. 2:**
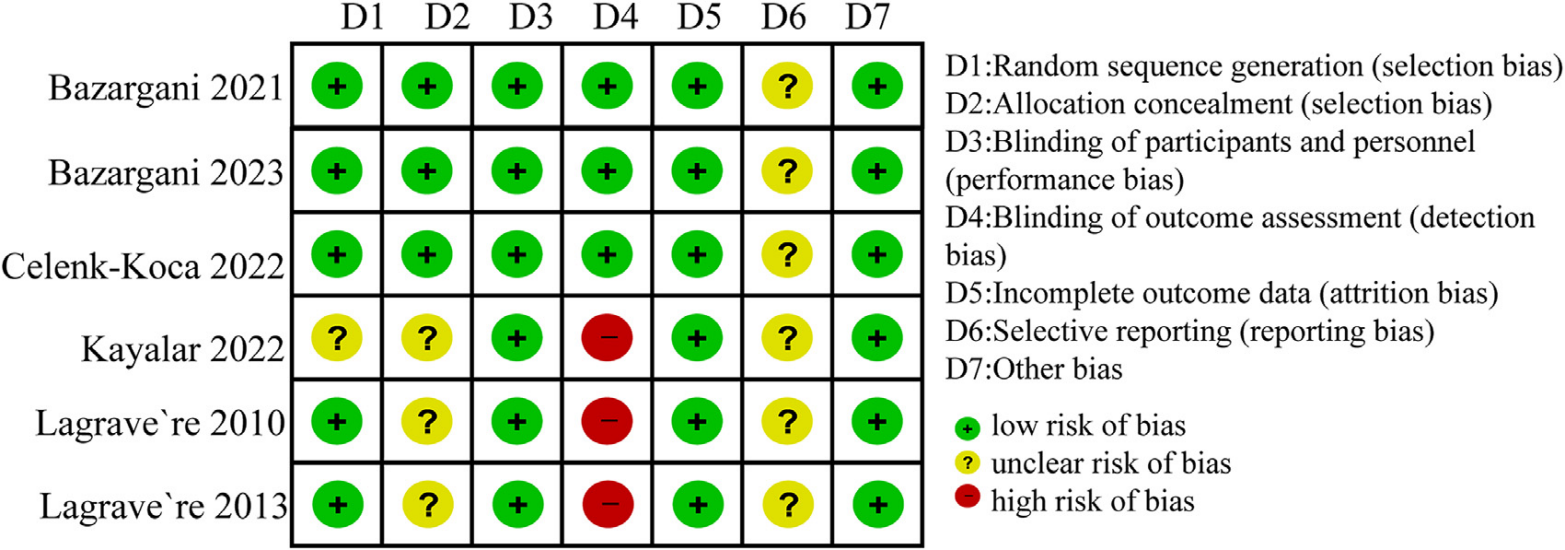
Risk of bias in included studies.

The risk of bias of the included RCTs was presented graphically in Figure 2 (Supplementary Table 2).

For the non-RCTs, three trials were judged to have moderate risk of bias, mainly because of missing data and measurement of outcome (Supplementary Table 3).^26^^,^^30^^,^^33^

The overall quality of available evidence using the GRADE statement was judged to be moderate for studies that analysed the dental maxillary expansion of TB appliances and BB appliances at 6 months postexpansion,^26^, ^27^, ^28^^,^^31^^,^^33^ moderate for studies that analysed the dental maxillary expansion of TB appliances and TBB appliances at 6 months postexpansion,^29^^,^^30^^,^^32^ and high for studies that analysed the skeletal maxillary expansion of TB appliances and BB appliances at 6 months postexpansion.^26^, ^27^, ^28^, ^29^, ^30^^,^^33^ Summary of findings table (SoF) for the GRADE statement is presented in Table 4.

**Table 4:** Summary of findings table (SoF) for GRADE statement of included studies.

Outcome	Risk of bias	Inconsistency	Indirectness	Imprecision	Publication bias	Grade level of evidence	Study
**Dental maxillary expansion (TB vs BB)**	Serious	Very serious	Very serious	Very serious	Very serious	⊕°°° Very low	Lagravère et al^28^; Celenk-Koca et al^31^; Mehta et al^26^; Altieri and Cassetta^33^
**Dental maxillary expansion (TB vs TBB)**	Serious	Very serious	Very serious	Very serious	Very serious	⊕°°° Very low	Garib et al^30^; Kayalar et al^29^
**Skeletal maxillary expansion (TB vs BB)**	Serious	Very serious	Serious	Serious	Very serious	⊕°°° Very low	Lagravère et al^28^; Mehta et al^26^; Altieri and Cassetta^33^

### Results of individual studies

#### Dental maxillary expansion

**Fig. 3:**
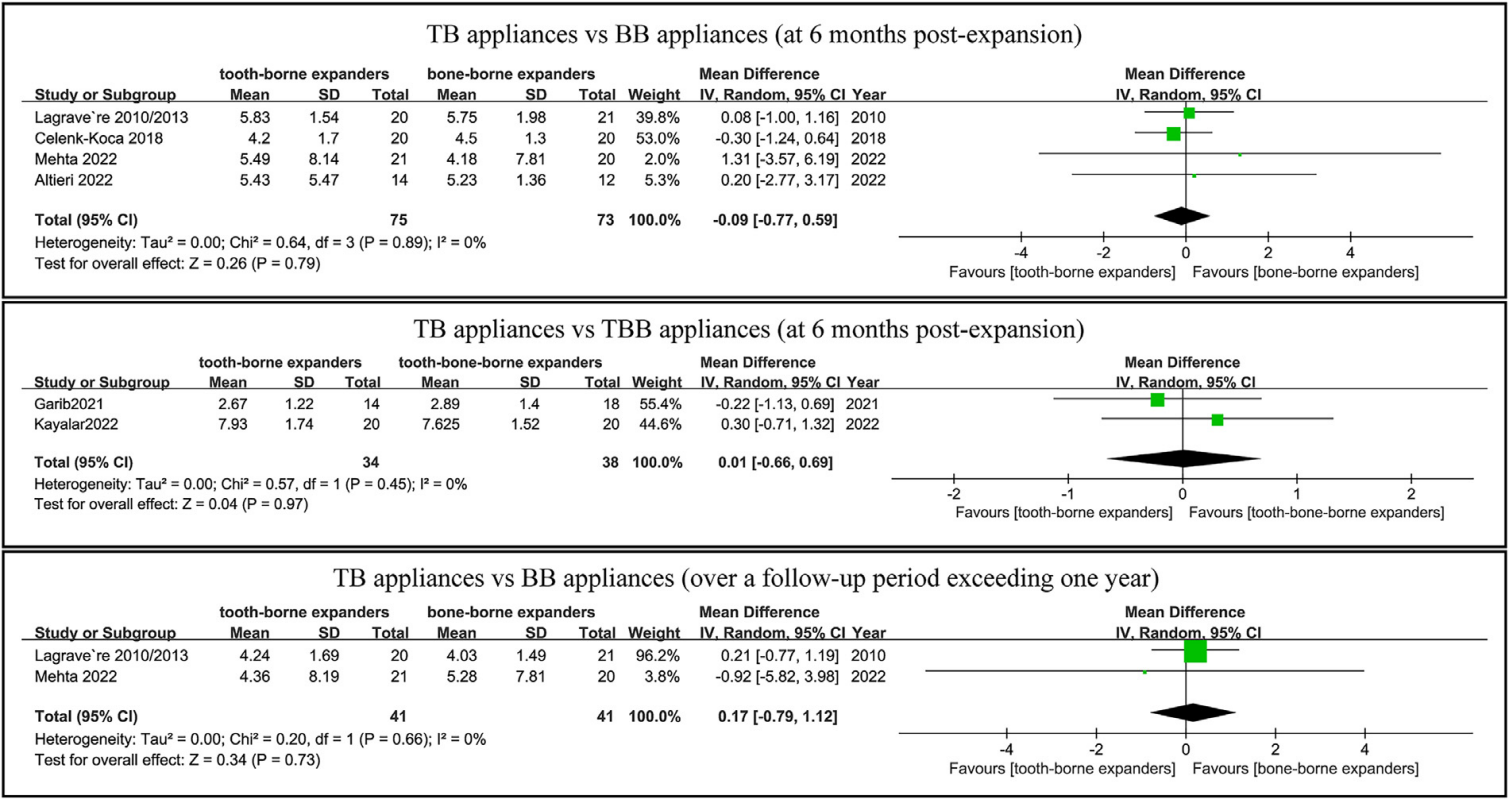
Forest plot comparing dental maxillary expansion in TB and BB/TBB appliances at follow-up periods of 6 months and more than 1 year.

The TB appliances demonstrated equal dental maxillary expansion at 6 months postexpansion when compared to the BB appliances in five studies involving 148 participants^26^, ^27^, ^28^^,^^31^^,^^33^ and the TBB appliances in two studies with 72 participants (Figure 3).^29^^,^^30^

Additionally, three studies showed equivalent dental maxillary expansion over a follow-up period exceeding 1 year (Figure 3).^26^, ^27^, ^28^ Bazargani et al^32^ showed that the dental maxillary expansion over a follow-up period exceeding 1 year was neither clinically nor statistically significant between the TB appliances and the TBB appliances.

#### Skeletal maxillary expansion

**Fig. 4:**
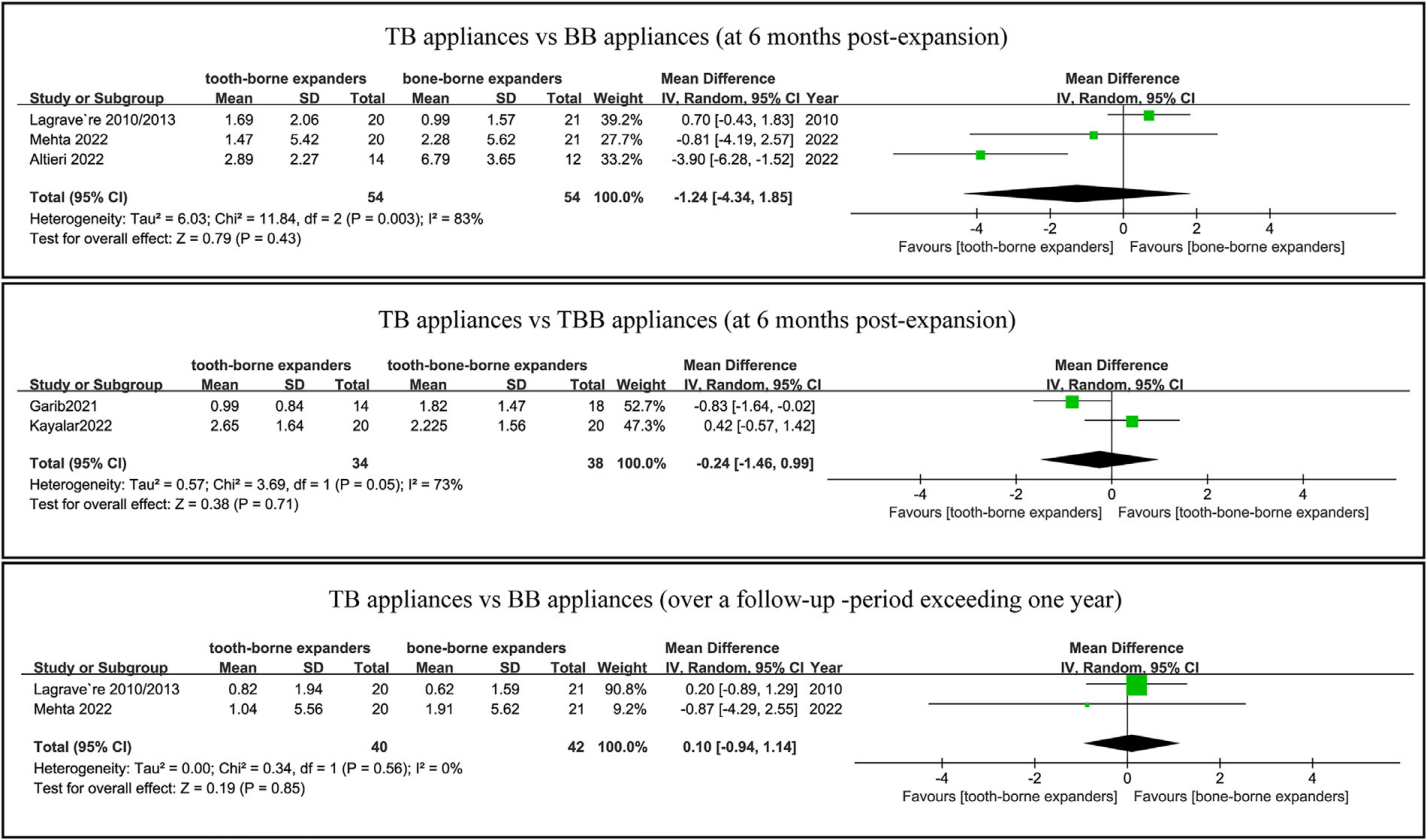
Forest plot comparing skeletal maxillary expansion in TB and BBTBB appliances at follow-up periods of 6 months and more than 1 year.

The TB appliances showed equal skeletal maxillary expansion at 6 months postexpansion, compared with both BB appliances in four studies with 108 participants,^26^, ^27^, ^28^^,^^33^ and TBB appliances in two studies with 72 participants.^29^^,^^30^ There was no statistical difference in the amount of maxillary expansion between the TB and BB appliances over a follow-up period exceeding 1 year (Figure 4).

#### Buccal bone thickness

The TB appliances had more buccal bone width loss and vertical bone height loss at 6 months postexpansion compared to the BB appliances.^26^^,^^31^^,^^33^ There are no significant difference between the TB appliances and BB appliances over a follow-up period exceeding 1 year.^26^

Bazargani et al^32^ found no significant difference of the marginal bone level at buccal aspects of first molars bilaterally between the TB appliances and TBB appliances over a follow-up period exceeding 1 year.

#### Molar inclination

Dental side effects were reported in eight articles.^26^, ^27^, ^28^, ^29^, ^30^, ^31^, ^32^, ^33^ A variety of different measurements were used.

The TB appliances had more molar inclination change than the BB appliances at 6 months postexpansion.^28^^,^^31^^,^^33^ However, the difference between the two appliances was not significant over a follow-up period exceeding 1 year.^26^^,^^28^ The TB appliances compared to the TBB appliances did not show a significant difference in molar inclination 6 months or longer after RME.^29^^,^^30^^,^^32^

## Discussion

### Summary of evidence

The aim of this systematic review and meta-analysis was to compare the long-term (≥6 months postexpansion) effects of TB appliances with BB or TBB appliances in RME, with a focus on evidence derived from three-dimensional assessment methods.

Selection bias was reduced, including all languages, years, and types of publications. All types of published and unpublished articles were also included with an extended search into seven databases, including grey literature. Through the inclusion of two independent authors in the research selection and the extraction of relevant data, risks and evidence quality assessments have reduce reviewer search and selection bias.

For this systematic review, the differences between TB and BB appliances were evaluated in five publications, whereas the differences between TB and TBB appliances were compared in two papers.

Regarding the primary outcome of this systematic review, namely dental maxillary expansion, there were no statistical differences between the TB and BB or TBB appliances at 6 months postexpansion and over a follow-up period exceeding 1 year. One previous review suggests that BB appliance may be associated with greater maxillary expansion compared with TB appliance, but their follow-up period was shorter.^10^

Our study found that after long-term postexpansion, different appliances produced similar skeletal effects, which is consistent with the study by Canan and Şenışık.^11^ Although adding bone support to RME devices can transfer forces directly to the palatal bone near the centre of resistance,^35^^,^^36^ providing greater skeletal expansion during expansion, the midpalatal suture opening is a triangular cross-section with the base located below and the apex pointing upward,^37^^,^^38^ which explains the lack of significant differences in skeletal maxillary expansion between the TB and BB/TBB appliances. This is different from previous findings and may also be attributed to long-term bone remodelling.^10^^,^^38^

The amount of expansion, age, postexpansion retention time, and overall treatment time are influential factors in the reduction in buccal bone thickness.^39^ In this study, meta-analysis for buccal bone thickness could not be performed due to the heterogeneity of measurement methods and reported outcomes. Two studies found that the use of the TB appliance resulted in a statistically significantly greater buccal bone resorption at 6 months postexpansion compared to the BB appliance.^31^^,^^33^ This was not the case in the other study, however.^26^ There was no statistically significant difference in buccal bone resorption between the groups as the buccal lateral bone plate recovered over a follow-up period exceeding 1 year.^26^^,^^40^ Caution is warranted in the interpretation of these findings, since this might be attributed to the limited number of trials with small sample sizes and heterogeneous results.

It is important to keep in mind the pyramidal or triangular shape of the suture opening due to the two centres of rotation, which leads to the bending of the alveolar bone and the subsequent tipping of the tooth.^11^^,^^41^ Although the study by Lagravère et al showed a slightly greater molar inclination of the BB appliances than the TB appliances at 6 months postexpansion.^28^^,^^42^ Most other studies showed a significantly greater change in molar inclination of the TB appliances than in the BB appliances^26^^,^^31^^,^^33^; Both articles showed similar molar inclination over a follow-up period exceeding 1 year.^26^, ^27^, ^28^ Garib et al^30^ showed no statistical difference in the amount of molar inclination between TB appliances and TBB appliances at 6 months postexpansion, but Kayalar et al^29^ showed a greater amount of molar inclination of the TB appliances at the early adolescent and late adolescent stages. The use of different appliances during treatment may lead to different degrees of tooth inclination. This should be taken into account when developing treatment plans to prevent unnecessary tipping.

In summary, this review found dental maxillary expansion and skeletal maxillary expansion are similar using different appliances in RME. No statistically significant differences were found in molar inclination and buccal bone resorption caused by different appliances during follow-up periods exceeding 1 year.

### Strengths and limitations

This systematic review has several strengths, including an a priori registered protocol, a comprehensive literature search^43^; the inclusion of randomized or matched nonrandomized studies; the robust review procedures; and the application of the GRADE approach to assess the strength of the provided recommendations.^44^

However, this review has important limitations that should be acknowledged. Most notably, the substantial clinical heterogeneity across studies in expansion protocols (0.2-1.0 mm/d), retention durations (immediate to 11 months), and follow-up timelines (6 months to 5 years) represents a significant challenge for data interpretation. While we employed random-effects models to address variability, the limited number of studies precluded meaningful stratified analyses based on these protocol differences. The decision to proceed with meta-analysis despite these limitations was based on the clinical relevance of addressing the overarching question of long-term effectiveness across real-world practice variations. The random-effects model provides a conservative approach to synthesizing this heterogeneous evidence. The evidence retrieved from this systematic review has to be interpreted with caution, as retrospective studies were included. When non-RCTs are used, selection bias may not be ruled out; inclusion of non-RCTs in meta-analyses is not considered prohibited, as long as a robust bias assessment is conducted and recent guidance on how to appropriately include such designs is provided.^45^ In addition, most meta-analyses are primarily based on small trials, which may affect the precision of estimates.^46^

## Conclusions

• There was no significant difference in dental maxillary expansion between the TB and BB/TBB appliances at follow-up periods of 6 months and more than 1 year.
• There was no significant difference in skeletal maxillary expansion between the TB and BB appliances at follow-up periods of 6 months and more than 1 year. Similarly, there was no significant difference between the TB and TBB appliances at 6 months postexpansion.
• Buccal bone resorption was greater with the TB appliances compared to BB appliances at 6 months postexpansion, but this different was not statistically significant at follow-up periods of more than 1 year. There was no difference in bone resorption in the TB appliances compared to the TBB appliances at follow-up periods of more than 1 year.
• Molar inclination was greater with the TB appliances compared to BB appliances at 6 months postexpansion, but this different was not statistically significant at more than 1 year postexpansion. There was no difference between the TB appliances and TBB appliances at follow-up periods of 6 months and more than 1 year.
• Despite considerable heterogeneity in study protocols, the overall evidence suggests comparable long-term effectiveness among different RME appliance types when assessed through three-dimensional methods.

## Registration and protocol

The PRISMA statement 2020 was used to develop the protocol of this systematic review, to compose the article, and for the compilation of the flowchart (PRISMA flow diagram). The protocol of this systematic review and meta-analysis was registered in the US National Institute of Health’s (NIH; Bethesda, Maryland, USA) International Prospective Register of Systematic Reviews (PROSPERO) research database (https://www.crd.york.ac.uk/prospero/; Trial Registration No. PROSPERO (CRD42023388623).

## Funding

This work was supported by the National Natural Science Foundation of China (82071080), the Shandong Natural Science Foundation (Grant No. ZR2024MH061), and the Fundamental Research Funds for the Central Universities (2022JC017).

## CRediT authorship contribution statement

**Jingyi Yu:** Conceptualization, Methodology, Software, Writing – original draft, Data curation. **Feng Qiu:** Conceptualization, Writing – original draft. **Mengfei Xu:** Data curation, Methodology. **Jixiao Wang:** Supervision, Writing – review & editing. **Lan Li:** Supervision, Writing – review & editing. **Fulan Wei:** Supervision, Writing – review & editing.

## Conflict of interest

The authors declare that they have no known competing financial interests or personal relationships that could have appeared to influence the work reported in this article. All authors read and approved the final version of this article.

## Appendix Supplementary materials

Image, application 1

Image, application 2

## Footnotes

Supplementary material associated with this article can be found in the online version at doi:10.1016/j.identj.2025.104024.
