# Supplementary material for: Long-Term Effects of Three Different Appliances for Rapid Maxillary Expansion: A Systematic Review and Meta-Analysis
Source: Int Dent J. 2025 Nov 20;76(1):104024. doi: 10.1016/j.identj.2025.104024 (PMC12671374; doi:10.1016/j.identj.2025.104024)
Supplement: Supplementary file 1 [file mmc1.doc]

**Supplementary Table 1 – Search strategy for electronic databases.**

| **Database** | **Search** | **Limits** | **Hits** |
| --- | --- | --- | --- |
| MEDLINE (via PubMed) | (((((((("Palatal Expansion Technique"[Mesh]) OR (Technique, Palatal Expansion[Title/Abstract])) OR (Technic, Palatal Expansion[Title/Abstract])) OR (Maxillary Expansion[Title/Abstract])) OR (Expansion, Maxillary[Title/Abstract])) OR (palatal expander[Title/Abstract])) OR (Maxilla Expansion[Title/Abstract])) OR (RME[Title/Abstract])) AND (((((((((((((((((((((("Orthodontic Anchorage Procedures"[Mesh]) OR (Procedures, Orthodontic Anchorage[Title/Abstract])) OR (Orthodontic Anchorage Technique[Title/Abstract])) OR (Technique, Orthodontic Anchorage[Title/Abstract])) OR (bone-anchorage[Title/Abstract])) OR (bone-anchored[Title/Abstract])) OR (bone-borne[Title/Abstract])) OR (implant-anchorage[Title/Abstract])) OR (implantanchored[Title/Abstract])) OR (Miniscrew[Title/Abstract])) OR (mini-screw[Title/Abstract])) OR (mini-implant[Title/Abstract])) OR (palatal implant[Title/Abstract])) OR (palatal distractor[Title/Abstract])) OR (skeletal anchorage[Title/Abstract])) OR (skeletally-anchored[Title/Abstract])) OR (transpalatal distraction[Title/Abstract])) OR (transpalatal distractor[Title/Abstract])) OR (tooth-borne[Title/Abstract])) OR (Tooth-anchored[Title/Abstract])) OR (tooth anchor[Title/Abstract])) OR (hyrax[Title/Abstract])) |  | 775 |
| Embase | #1'palatal expansion'/exp OR 'palatal expansion technique':ab,ti,kw OR 'technique, palatal expansion':ab,kw,ti OR 'technic, palatal expansion':ab,kw,ti OR 'maxillary expansion':ab,kw,ti OR 'expansion, maxillary':ab,kw,ti OR 'palatal expander':ab,kw,ti OR 'maxilla expansion':ab,kw,ti OR 'rme':ab,kw,ti #2'orthodontic anchorage'/exp OR 'orthodontic anchorage procedures':ab,kw,ti OR 'procedures, orthodontic anchorage':ab,kw,ti OR 'orthodontic anchorage technique':ab,kw,ti OR 'technique, orthodontic anchorage':ab,kw,ti OR 'bone-anchorage':ab,kw,ti OR 'bone-anchored':ab,kw,ti OR 'bone-borne':ab,kw,ti OR 'implant-anchorage':ab,kw,ti OR 'implantanchored':ab,kw,ti OR 'miniscrew':ab,kw,ti OR 'mini-screw':ab,kw,ti OR 'mini-implant':ab,kw,ti OR 'palatal implant':ab,kw,ti OR 'palatal distractor':ab,kw,ti OR 'skeletal anchorage':ab,kw,ti OR 'skeletally-anchored':ab,kw,ti OR 'transpalatal distraction':ab,kw,ti OR 'transpalatal distractor':ab,kw,ti OR 'tooth-borne':ab,kw,ti OR 'tooth-anchored':ab,kw,ti OR 'tooth anchor':ab,kw,ti OR 'hyrax':ab,kw,ti #1AND#2 |  | 574 |
| Web of science | Same as Embase |  | 694 |
| Cochrane | Same as Embase | Not Reviews | 208 |
| Scopus | Same as Embase |  | 831 |
| Proquest | Same as Embase |  | 208 |
| Science direct | ("Orthodontic Anchorage Procedures " OR " bone-anchored " OR " Miniscrew " OR " Tooth-anchored " OR " hyrax ") AND ("Palatal Expansion Technique " OR " Maxilla Expansion " OR " palatal expander " OR " RME") |  | 71 |
| Clinical trils.gov | ("maxillary expansion" OR ((expand* OR expans*) AND maxill*)) |  | 41 |
| SUM (with overlap) | | | 3402 |
