# Supplementary material for: Long-Term Effects of Three Different Appliances for Rapid Maxillary Expansion: A Systematic Review and Meta-Analysis
Source: Int Dent J. 2025 Nov 20;76(1):104024. doi: 10.1016/j.identj.2025.104024 (PMC12671374; doi:10.1016/j.identj.2025.104024)
Supplement: Supplementary file 2 [file mmc2.doc]

**Supplementary Table 2.** Risk of bias assessment of included randomized trials.

| **author** | **Random sequence generation** | **Allocation concealment** | **Blinding of participants and personnel** | **Blinding of outcome assessment** | **Incomplete outcome data** | **Selective reporting** | **Other sources of bias** | **Over all** |
| --- | --- | --- | --- | --- | --- | --- | --- | --- |
| Lagrave`re 2010/ 2013 | Low risk –" ...the subjects were randomized into the groups by using a random numbers generated list." | Unclear – No mention throughout the paper. | Low risk - Blinding of participants and personnel impossible; outcome is objective and has been assessed and analyzed blindly. | High risk – No mention of blinding throughout the paper; blinding however is possible. | Low risk – no drop outs. | Unclear – no obvious selective reporting; however, it is difficult to judge however whether selective reporting is a problem, as no protocol exists. | Low risk – no other sources of bias; intervention is dependent on patient compliance (which is not reported), but as treatment end was based on objective effects (skeletal and dentoalveolar effects), this was considered irrelevant. | High risk |
| Lagrave`re 2013 | Low risk –" ...the subjects were randomized into the groups by using a random numbers generated list." | Unclear – No mention throughout the paper. | Low risk - Blinding of participants and personnel impossible; outcome is objective and has been assessed and analyzed blindly. | High risk – No mention of blinding throughout the paper; blinding however is possible. | Low risk – no drop outs. | Unclear – no obvious selective reporting; however, it is difficult to judge however whether selective reporting is a problem, as no protocol exists. | Low risk – no other sources of bias; intervention is dependent on patient compliance (which is not reported), but as treatment end was based on objective effects (skeletal and dentoalveolar effects), this was considered irrelevant. | High risk |
| Bazargani 2021/ 2023 | Low risk –" The randomization procedure was as follows: a computer-generated randomization list was as follows: a computer-generated randomization list..." | Low risk –"...and stored with a research secretary. Each time a patient gave his/her consent, the secretary was contacted by email to provide the information about which type of expander the patient should receive. " | Low risk - Blinding of participants and personnel impossible; outcome is objective and has been assessed and analyzed blindly. | Low risk – "Due to clinical limitations, only the outcome assessors were blinded to the groups to which the patients were allocated." | Low risk – low drop-out rate (4%) which is transparently reported and balanced:" One patient in each group missed a CBCT appointment at T1" | Unclear – no obvious selective reporting; however, it is difficult to judge however whether selective reporting is a problem, as no protocol exists. | Low risk – no other sources of bias; intervention is dependent on patient compliance (which is not reported), but as treatment end was based on objective effects (Skeletal and dentoalveolar effects), this was considered irrelevant. | Unclear |
| Bazargani 2023 | Low risk –" The randomization procedure was as follows: a computer-generated randomization list was as follows: a computer-generated randomization list..." | Low risk –"...and stored with a research secretary. Each time a patient gave his/her consent, the secretary was contacted by email to provide the information about which type of expander the patient should receive. " | Low risk - Blinding of participants and personnel impossible; outcome is objective and has been assessed and analyzed blindly. | Low risk – "Due to clinical limitations, only the outcome assessors were blinded to the groups to which the patients were allocated." | Low risk – low drop-out rate (4%) which is transparently reported and balanced:" One patient in each group missed a CBCT appointment at T1" | Unclear – no obvious selective reporting; however, it is difficult to judge however whether selective reporting is a problem, as no protocol exists. | Low risk – no other sources of bias; intervention is dependent on patient compliance (which is not reported), but as treatment end was based on objective effects (Skeletal and dentoalveolar effects), this was considered irrelevant. | Unclear |
| Kayalar 2022 | Unclear –"All of the CBCT images were obtained from two previous randomised controlled clinical trials..." | Unclear – No mention throughout the paper. | Low risk - Blinding of participants and personnel impossible; outcome is objective and has been assessed and analyzed blindly. | High risk – No mention of blinding throughout the paper; blinding however is possible. | Low risk – no drop outs. | Unclear – no obvious selective reporting; however, it is difficult to judge however whether selective reporting is a problem, as no protocol exists. | Low risk – no other sources of bias; intervention is dependent on patient compliance (which is not reported), but as treatment end was based on objective effects (skeletal, dentoalveolar, and buccal bone changes), this was considered irrelevant. | High risk |
| Celenk-Koca 2018 | Low risk – "Patients were randomly assigned to one of the two treatment groups via a block randomization procedure with a block size of four, using a computer-generated list of random numbers". | Low risk – “The allocation sequence was concealed from the orthodontist, researchers, and the patients. When a patient was deemed as eligible for enrollment, the patient was assigned to a treatment group using opaque and sealed envelopes containing the allocation number.” | Low risk - Blinding of participants and personnel impossible; outcome is objective and has been assessed and analyzed blindly. | Low risk – "Since it was impossible to blind the patient and orthodontist to the treatment groups, the researcher who traced the cone-beam computed tomography (CBCT) images and the statistician who evaluated the data were blinded." | Low risk – no drop outs. | Unclear – no obvious selective reporting; however, it is difficult to judge however whether selective reporting is a problem, as no protocol exists. | Low risk – no other sources of bias; intervention is dependent on patient compliance (which is not reported), but as treatment end was based on objective effects (expansion), this was considered irrelevant. | Unclear |

**Supplementary Table 3.** Risk of bias assessment of included non-randomized trials.

| **Study** | **Confounding** | **Selection of participants** | **Classification of intervention** | **Deviation from intended intervention** | **Missing data** | **Measurement of outcome** | **Selection of reported results** | **Overall** |
| --- | --- | --- | --- | --- | --- | --- | --- | --- |
| Mehta et al.2022 | Low | Low | Low | Low | Low | Moderate | Low | Moderate |
| Garib et al.2021 | Low | Low | Low | Low | Moderate | Low | Low | Moderate |
| Altieri et al.2022 | Low | Low | Low | Low | Moderate | Moderate | Low | Moderate |
